# Integrated Process for *Schizochytrium* Oil Extraction, Enzymatic Modification of Lipids and Concentration of DHA Fatty Acid Esters Using Alternative Methodologies

**DOI:** 10.3390/md22040146

**Published:** 2024-03-26

**Authors:** Gonzalo Berzal, Paz García-García, Francisco Javier Señoráns

**Affiliations:** Healthy-Lipids Group, Food Science Department, Faculty of Sciences, Universidad Autónoma de Madrid, Francisco Tomás y Valiente, 7, 28049 Madrid, Spain; gonzalo.berzal@inv.uam.es (G.B.); mariap.garcia@uam.es (P.G.-G.)

**Keywords:** microalgae, *Schizochytrium* sp., DHA, Novozym^®^435, pressurized liquid extraction, enzymatic ethanolysis

## Abstract

Marine microalgae *Schizochytrium* sp. have a high content of docosahexaenoic acid (DHA), an omega-3 fatty acid that is attracting interest since it prevents certain neurodegenerative diseases. The obtention of a bioactive and purified DHA fatty acid ester using a whole-integrated process in which renewable sources and alternative methodologies are employed is the aim of this study. For this reason, lyophilized *Schizochytrium* biomass was used as an alternative to fish oil, and advanced extraction techniques as well as enzymatic modification were studied. Microalgal oil extraction was optimized via a surface-response method using pressurized liquid extraction (PLE) obtaining high oil yields (29.06 ± 0.12%) with a high concentration of DHA (51.15 ± 0.72%). Then, the enzymatic modification of *Schizochytrium* oil was developed by ethanolysis using immobilized *Candida antarctica* B lipase (Novozym^®^ 435) at two reaction temperatures and different enzymatic loads. The best condition (40 °C and 200 mg of lipase) produced the highest yield of fatty acid ethyl ester (FAEE) (100%) after 8 h of a reaction attaining a cost-effective and alternative process. Finally, an enriched and purified fraction containing DHA-FAEE was obtained using open-column chromatography with a remarkably high concentration of 93.2 ± 1.3% DHA. The purified and bioactive molecules obtained in this study can be used as nutraceutical and active pharmaceutical intermediates of marine origin.

## 1. Introduction

In recent years, several studies have focused on polyunsaturated fatty acids (PUFA), specially omega-3, and their beneficial effects on health [1,2]. In particular, docosahexaenoic acid (DHA, C22:6 *n*-3), a long-chained PUFA (LC-PUFA) [3], is being investigated due to its capability to modulate many inflammatory processes and its key role in brain development [4]. Thus, DHA is proposed to prevent neurodegenerative diseases, such as Alzheimer’s or Parkinson’s, as well as inflammatory diseases [5,6,7].

Nevertheless, to efficiently increase the amount of DHA present in neuronal tissues, DHA may be incorporated in the diet via nutritional supplements and nutraceuticals, since conversion from α-linolenic acid to DHA in adults is limited [8,9]. In this way, the preferred sources of DHA are fish and krill, but microalgae have emerged as an alternative and ecological source of DHA [8,10] that is in agreement with the sustainability of marine resources. Therefore, bioactive compounds from microalgal oils are gaining importance as a renewable and sustainable source of DHA [8,11,12].

Among different microalgae that are harvested worldwide and accepted as novel food, *Schizochytrium* sp. is emphasized due to its high lipid content, especially regarding the high amounts of DHA (around 40% DHA), whose accumulation depends on culture conditions [13,14]. This microalgal oil is advantageous in the industry because of its heterotrophic culture conditions, which enable elevated DHA production in the form of triacylglycerols (TAG) [15]. Moreover, *Schizochytrium* oil is also composed of three other principal fatty acids, myristic (C14:0), palmitic (C16:0), and docosapentaenoic acid (C22:5 n-6, DPA), the main acids found in its composition [13,14]. For their extraction, modern technologies such as pressurized liquid extraction (PLE) arise as an alternative to traditional extraction procedures [16,17]. PLE uses high temperatures and pressures that can extract bioactive compounds extremely quickly, avoiding their oxidation and deterioration in a process that follows the principles of green chemistry [18,19,20,21,22]. Thus, the possibility of modifying these parameters enables the optimization of lipid extraction for its consequent hydrolysis by lipases.

Lipases (EC 3.1.1.3) are used in a wide range of industrial applications [23,24,25] in the pharmaceutical and food industries. Over the last decade, oil hydrolysis and transesterification by lipases have become more important [26,27,28]. However, only a few reports focus on the ethanolysis of microalgae oil using lipases [29,30,31,32]. The esterification of LC-PUFA, such as DHA, is developed by immobilized lipases under mild conditions to maintain the labile structure of the fatty acid. The use of immobilized lipases as industrial biocatalysts is the most suitable method for developing more selective, controlled, and rapid procedures in the industry [33,34,35,36]. Furthermore, immobilization allows for the industrial reuse of the biocatalyst for several cycles as a result of an increase in its stability, and the easy separation of the desired product, obtaining cost-efficient procedures [37,38,39,40].

*Candida antarctica* lipase B (CALB) is an immobilized lipase, commercially known as Novozym^®^435, characterized by its versatility, high activity, and stability [41]. CALB is used for oil modification, mainly in biodiesel production, as well as food industry [42]. It is able to cause oil ethanolysis in solvent-free systems according to green chemistry [26]. The molecules produced in the reaction (fatty acid ethyl esters, FAEE) [29] could serve as a scaffold to develop structured lipids enriched in DHA with improved properties and composition [1,43,44,45,46,47] using regioselective lipases [48,49,50].

In this study, a novel strategy to obtain an enriched DHA oil from *Schizochytrium* sp*.* was proposed. For this aim, different extraction conditions using PLE technology were compared and optimized, and the extracted oil was characterized by GC-MS. The enzymatic ethanolysis of microalgal oil was developed using CALB Novozym^®^435 to produce FAEE with the highest concentration of DHA, which can serve as a food supplement or nutraceutical for structured phospholipid synthesis to prevent neurodegenerative diseases. Thus, the hypothesis of this paper was to determine the possibility of producing DHA concentrates from sustainable raw materials as microalgae using environmentally friendly technologies.

## 2. Results and Discussion

### 2.1. Lipid Extraction from Schizochytrium sp. by Pressurized Liquids Compared to the Soxhlet Method

Lipid extraction of *Schizochytrium* lipids was compared using two different methods. On the one hand, Soxhlet was used as the traditional procedure for obtaining a reference lipidic yield. On the other hand, PLE was proposed as an alternative method for the advanced and fast extraction of bioactive compounds preserving the bioactivity.

The result obtained with Soxhlet was an extraction yield of 24.04 ± 0.25%. Lipid extraction using pressurized liquids was optimized using surface-response methodology. Parameters such as temperature and solvent polarity (hexane and ethanol) were evaluated in a previous study on *Nannochloropsis* lipids [51]. In this study, hexane was used as a non-polar solvent, ethanol as a polar solvent and a mixture of both with mild polarity (1:1) for lipid extraction, as reported for other microalgae species [52]. Moreover, different extraction times (5, 10 and 15 min) and different temperatures (from 80 to 120 °C) were also evaluated.

**Figure 1:**
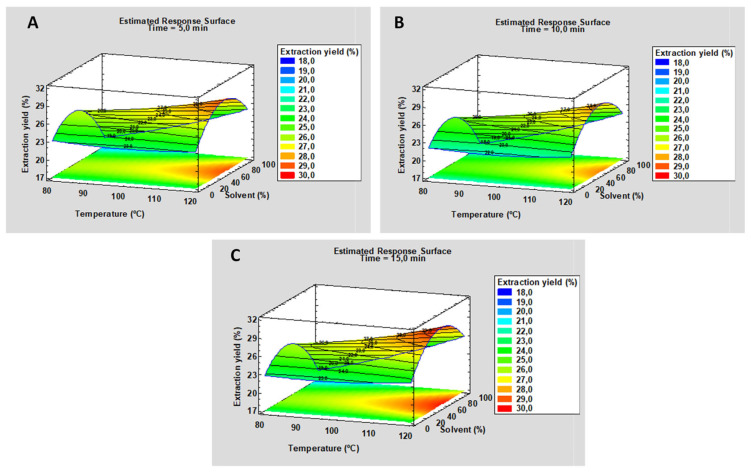
Surface-response plots obtained with the Statgraphics 19 program with different extraction times. (**A**) Corresponds to a static extraction time of 5 min. (**B**) Corresponds to a static extraction time of 10 min. (**C**) Corresponds to a static extraction time of 15 min.

Using the Statgraphics 19 program, the surface-response plots shown in Figure 1 were drawn.

As can be seen, the influence of different solvent mixtures on the extraction was revealed. Thus, using hexane and ethanol in equal parts (1:1), the yield improved compared to extraction only with hexane or ethanol for all the times studied. With respect to the static extraction time, there was no difference in the oil yield when this parameter was modified (Appendix A). In addition, an increase in the extraction yield was obtained, as previously found in the literature, with oil yields ranging from 17.13 ± 1.17% to a maximum yield of 29.06 ± 0.12% at 120 °C for 15 min, and with a hexane–ethanol 1:1 solvent mixture.

Thus, the extraction with a mixture of hexane–ethanol in a 1:1 proportion was used to scale up the method, with a final volume of 70 mL of solvent. In this case, a 26.15 ± 1.13% extraction yield was achieved, notably similar to that obtained by the standard protocol with less volume. In the scale-up, a solvent saving system was configured with up to 10 times the amount of microalgae. In both cases, the results found were also comparable to the yield found using the Soxhlet method, representing the valuable alternative of PLE against traditional methods, decreasing both the amount of non-friendly solvents, such as hexane, and the necessary time and energy. Therefore, the scale-up of the extraction method was useful as it allowed a higher amount of microalgal oil to be produced, leading to the development of further experiments of enzymatic modification.

### 2.2. Characterization of Schizochytrium sp. Oils

Starting with the HPLC-ELSD characterization (see Section 3.7), all oils used for the experimental development were analyzed, and the results show that they contained only TAG in their composition (see Appendix B), in accordance with the consulted literature [53].

For characterization of fatty acids of the TAG by GC-MS, an analysis of *Schizochytrium* sp*.* commercial oil and microalgae oils obtained using PLE in our laboratory was carried out. To compare their fatty acid profiles, as oils contained only TAG in their composition (no free fatty acids), their characterization in a basic medium is an effective method (see Section 3.5). GC-MS was employed to obtain different fractions of decreasing polarity and separate the fatty acid methyl esters. Fatty acids such as myristic (14:0), palmitic (16:0), stearic (18:0), oleic (18:1), linoleic (18:2), arachidonic (20:4), eicosapentaenoic (EPA), docosapentaenoic (DPA *n*-3 and *n*-6), and docosahexaenoic (DHA) were found.

According to results shown in Table 1, there were some differences between commercial oil and oils extracted using PLE with different solvents. In commercial oil a lack of myristic acid was found in the fatty acid profile, despite the fact that myristic acid is commonly found in other oils of *Schizochytrium* sp*.* described in the literature [13]. In our case, a ratio of 14:0 fatty acids was present in all microalgae oils obtained using PLE in around 10–13% of the total fatty acid composition.

Moreover, the composition of palmitic acid was similar in all studied oils (around 20% of total composition) as well as DPA *n*-6 (around 15% in all cases). Even though the DHA percentage in the composition was higher in the commercial oil, there was an impact on the whole fatty acid profile, as it did not have myristic oil. Therefore, DHA representativeness was diverse, and the results cannot be directly compared. Accordingly, the oil with the highest amount of DHA from all extracted oils tested was that produced by PLE with 1:1 hexane–ethanol. More than 50% of the total fatty acid composition corresponded to DHA, exceeding that extracted by Soxhlet by 5%. This result may be due to the methodology applied, since the Soxhlet method uses high temperatures for several hours. In the case of PLE, the sample is in an environment free of oxygen and light, unlike the Soxhlet method, so there is no oxidation of PUFA during short time extraction, nor when high temperatures such as 120 °C are used [54,55,56].

Regarding the fatty acid composition of *Schizochytrium* sp*.* oils extracted with different solvent mixtures, the extraction by PLE influenced extraction yield (see Section 2.1), but it did not have much impact on the fatty acid composition of the extracted oil.

### 2.3. Enzymatic Ethanolysis of Schizochytrium Oil

To optimize the enzymatic ethanolysis reactions of *Schizochytrium* oil and produce ethyl esters of the fatty acids, reaction kinetics at different temperatures (30 °C and 40 °C) and different loads of commercial lipase Novozym^®^ 435 (CALB) were studied.

**Figure 2:**
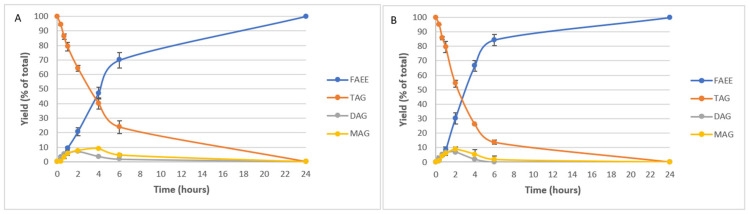
Kinetics of enzymatic ethanolysis, using CALB at 30 °C (**A**) and 40 °C (**B**), both at 200 rpm.

The results represented in Figure 2 show that the conversion of TAG into FAEE was similar at both reaction temperatures of 30 °C or 40 °C. However, remarkable results were obtained for the first two hours of reaction, where large differences between temperatures in the conversion of the initial TAG were observed. In this case, at 30 °C (Figure 2A) there was a 64.35% TAG compared to 20.68% FAEE. Comparing these results with those obtained at 40 °C (Figure 2B), at the same time as the reaction, there was 54.24% of TAG versus 30.18% of FAEE.

From this point, there was a higher conversion in FAEE at 40 °C than at 30 °C, while after 6 h of ethanolysis, at 40 °C, the yield of FAEE reached 84.47%; the yield at 30 °C was 69.84%. When considering the 24-h aliquot, in both cases, a yield of 100% was achieved in the conversion of TAG into FAEE. In all cases, no relevant differences in reaction intermediates (DAG and MAG) were observed, either at 30 °C or 40 °C.

Therefore, the increase in temperature favors the course of the enzymatic reaction, as was already discussed in the literature on Novozym^®^ 435 lipase [30]. Subsequently, enzymatic ethanolysis was also performed at 40 °C by adding twice the amount of the enzyme to study the influence of this parameter.

**Figure 3:**
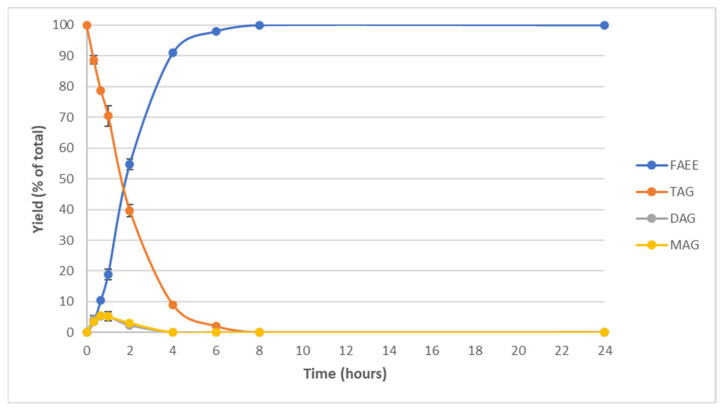
Kinetics of enzymatic ethanolysis with a double load of CALB at 40 °C and 200 rpm.

As seen in Figure 3, the reaction with a double load of CALB evolved more rapidly than the reaction with a normal load at the same temperature.

After 4 h, a yield of FAEE of 66.58% was achieved by adding the normal enzymatic load (Figure 2B). In comparison, when adding a double amount of enzyme, the yield obtained was 91.03%. Moreover, 100% FAEE was reached after only 8 h of reaction with a double enzymatic load, while it took 24 h to obtain this yield with a simple load. Therefore, by adding twice the enzymatic load at 40 °C and 200 rpm, 100% FAEE was produced after 8 h, so the time was considerably reduced, but more enzyme was used.

With this process, *Schizochytrium* oil composed by TAG was enzymatically modified in a mild process at 40 °C to produce FAEE that can be separated to produce concentrates of DHA for different purposes.

### 2.4. Open-Column Chromatography

Therefore, open-column chromatography was performed to separate and purify DHA from the rest of FAEE produced in the enzymatic ethanolysis described before. The sample was divided into different fractions that were eluted with different mixtures of solvents.

**Figure 4:**
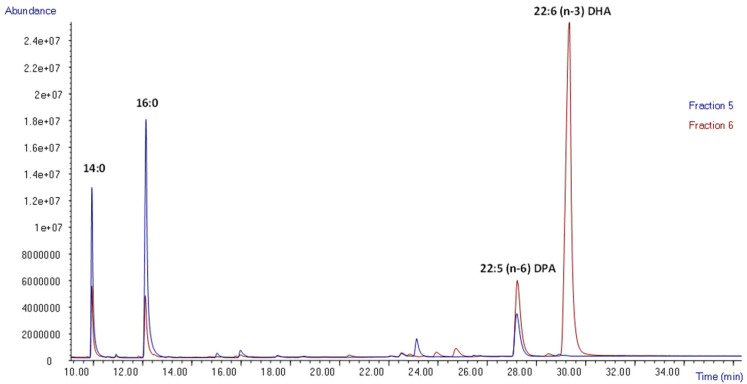
Analysis of FAEE profiles in fraction 5 (blue) and fraction 6 (red) obtained by open-column chromatography using GC-MS.

The first four fractions were discarded as FAEE was kept inside the column because of its affinity. A second round of hexane–ethyl acetate (95:5), which correspond to fraction 5 (F5) was needed for FAEE elution. F5 was analyzed by GC-MS and the corresponding chromatogram is represented in Figure 4 (blue). It can be seen that a large amount of saturated fatty acids (myristic acid and palmitic acid), as well as squalene and a low amount of DPA, were eluted.

Then, fraction 6 (F6) was eluted with hexane–ethyl acetate in proportion 90:10 and analyzed by GC-MS (Figure 4, red). In this case, the following values of FAEE were obtained: 5.0 ± 0.8% myristic acid, 6.3 ± 1.3% palmitic acid, 1.7 ± 0.1% EPA, 14.9 ± 0.1% DPA, and 72.0 ± 2.2% DHA. The initial proportion of fatty acids is explained in the characterization of microalgal oil 2 (see Section 2.2). Overall, a fraction with almost 90% of LC-PUFA was isolated, constituting mostly DHA. Moreover, in the next fraction of open-column chromatography (F7), there was not any peak corresponding to FAEE. Therefore, all the DHA extracted from *Schizochytrium* sp. was eluted in fraction 6, with a maximum recovery.

In order to attain a DHA-enriched fraction, the protocol of open-column chromatography was slightly modified as expressed in Section 3.9. The first four fractions were discarded. Moreover, either P5 or P6 of the purification protocol (duplicates) were composed of myristic and palmitic acid, squalene, a large part of DPA, and a low fraction of DHA. Subsequently, modifying the relation between hexane and ethyl acetate from 95:5 to 92:8 and using two cycles of elution under the same conditions enabled the total elution of certain FAEE. However, part of DHA was also eluted in this fraction, avoiding the full recovery of DHA.

**Figure 5:**
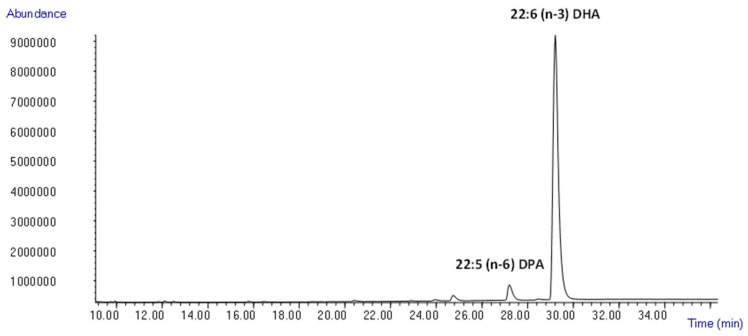
Analysis of FAEE profile in an enriched DHA fraction isolated by purification in open column chromatography using GC-MS.

Furthermore, in the last step of purification, hexane–ethyl acetate in proportion 90:10 was used. This protocol was also carried out in duplicate, obtaining a fraction with the following values of FAEE: 2.0 ± 0.4% EPA, 4.8 ± 0.9% DPA, and 93.2 ± 1.3% DHA (Figure 5). In this case, a purity higher than 90% DHA was achieved.

Hence, a fraction with 75.0 ± 14.9 mg with a purity of 93.2% of DHA was obtained from 0.3 g of FAEE that was introduced in the open column and produced by enzymatic ethanolysis. If the recovery of each of the duplicates was calculated in terms of average and standard deviation, a yield of 44.72 ± 6.99% DHA was obtained. Taking into account that the percentage of DHA extracted with PLE using hexane–ethanol (1:1) was 51.2% of the total fatty acid composition, almost 90% of DHA was recovered from *Schizochytrium* sp*.* with a high purity.

## 3. Materials and Methods

### 3.1. Materials

Lyophilized *Schizochytrium* sp. biomass was provided by Cianoalgae S.L. (Madrid, Spain). *Schizochytrium* sp. commercial oil was acquired from Nutilab S.L. (Valencia, Spain). The oil and microalgae were kept at cool temperatures and protected from light to avoid oxidation. Commercial immobilized lipase Novozym^®^ 435 (Candida Antarctica B lipase, CALB) was kindly donated from Novozymes A/S (Bagsvaerd, Denmark). Hexane, ethyl acetate, and methyl tert-butyl ether (MTBE) were purchased from Avantor Performance Materials (Gliwice, Poland). Molecular sieves (3Å) were purchased from Scharlab S.L. (Sentmenat, Spain). Methanol, acetone, and isopropanol (HPLC-grade) were provided by Lab-Scan Analytical Sciences (Gliwice, Poland). Ethanol and reagents of common use were provided by Panreac Quimica S.A.U. (Barcelona, Spain).

### 3.2. Traditional Lipid Extraction by Soxhlet Method

For Soxhlet extraction [57], 4.0 g of lyophilized *Schizochytrium* sp. was used, and 150 mL of hexane was added in a Soxhlet extractor with continuous boiling and condensation cycles of the solvent for 6 h (matrix/solvent ratio 1:37.5). Subsequently, the extracts were evaporated with a rotary evaporator (Heidolph Hei-Vap Value HB/G3, Germany, Berlin) at 35 °C followed by nitrogen stream to constant weight. Lipid content was determined gravimetrically and calculated as percentage by weight of dry biomass (Equation (1)).

(1) $$Lipid\;content\left(\%\right)=\frac{Evaporated\;extract\;weight(mg)}{Dry\;biomass\;weight(mg)}\times \;100$$

The extracts obtained were stored in a nitrogen atmosphere in the dark at 4 °C until analysis. In all cases, the experiments were carried out at least in triplicate.

### 3.3. Pressurized Liquid Extraction of Microalgal Biomass

PLE was carried out with a DIONEX ASE 350 extractor (Sunnyvale, California) equipped with stainless steel extraction cells (10 mL volume). Lyophilized *Schizochytrium* sp. was weighed (1 g) and loaded into the extraction cell, adding 2 g of sand at both ends of the cell.

The extraction cell was then filled with the different solvents used: hexane, ethanol, and a mixture of both (1:1), different temperatures (80, 100, and 120 °C) and different static extraction times (5, 10, and 15 min) were also tested. For this purpose, a surface-response experimental design was created by introducing all of the variables mentioned above, using the Statgraphics 19 statistical program. The volume of solvent used was 20–25 mL, depending on cell temperature and pressure. Finally, the extract was recovered under a stream of nitrogen in 50 mL vials and kept at 4 °C until analysis. Each of the extraction conditions was performed in duplicate [51].

### 3.4. Scale-up of Pressurized Liquid Extraction

The extraction procedure was scaled up using 100 mL stainless steel cells. In this case, 20.00 g of lyophilized *Schizochytrium* sp. was weighed and loaded into the extraction cell, adding sand at both ends of the cell. A solvent saving mode with a flow rate of 1.5 mL/min was used. The static extraction time was 15 min and the volume of the solvent used was 69–75 mL. The scale-up was performed in triplicate.

### 3.5. Characterization of Schizochytrium sp. Oils by GC-MS

The fatty acid profile of *Schizochytrium* sp. oil was determined in duplicate, both from commercial oil and from different extractions carried out by PLE. Derivatization in a basic medium was developed following ISO TC34/SC 5 standard method (See Appendix C for explanation) [58]. The obtained fatty acid methyl esters (FAME) were analyzed by gas chromatography coupled to a mass spectrometer (GC-MS) using 100 µL of FAMEs and 400 µL of hexane for GC-MS analysis.

The fatty acid analysis was performed on an Agilent Technologies (Palo Alto, Cal., USA) 5975 MSD Series gas-mass chromatograph with an automatic injector and He as the carrier gas. An Agilent Technologies HP-88 capillary column was used, with dimensions of 100 m x 0.25 mm x 0.20 μm. The injection temperature was 250 °C. The oven was kept at 175 °C for 8 min. The temperature was then raised at a rate of 3 °C/min to 230 °C, which was maintained for an additional 10 min. The temperature of the detector was 230 °C. The amount of sample injected was 1 μL with a 1:20 split. The mass spectrometer used an ionization potential of 70 eV and an atomic mass range from 30 to 400 μ (atomic mass units).

The fatty acids were identified by comparing their retention times and the mass spectra (NIST Mass Spectral Library Version 2.0) with those obtained from the standards, expressing the amounts as percentages of the total FA content. The findings were compared with updated bibliographic references.

### 3.6. Enzymatic Ethanolysis of Schizochytrium sp. Oil

First, 200 mg of 3Å molecular sieves, which remove water to prevent hydrolysis reactions, and 100 mg of commercial CALB were weighed and placed in a 30 mL capacity glass vial. Then, 2.05 mL of hexane, 150 µL of absolute ethanol, and 300 µL of *Schizochytrium* sp. oil extracted by PLE were incorporated. Both solvents were dehydrated when in contact with molecular sieves. The reaction was carried out in a Heidolph incubator equipped with a platform shaker (Unimax 1010) and a heating unit, with constant agitation (200 rpm) at different temperatures (30 °C and 40 °C). The reaction was also tested at 40 °C with a double enzymatic load, according to the previously described method.

To study the reaction kinetics, 25 μL of the suspension (reaction medium) was taken at different times (0, 20 min, 40 min, 1 h, 2 h, 4 h, 6 h and 24 h), diluted 25 times in hexane and analyzed by high-performance liquid chromatography coupled with a light-scattering detector (HPLC-ELSD). All reactions were conducted in duplicates.

### 3.7. Analysis by HPLC-ELSD

HPLC-ELSD analysis was performed using an Agilent 1260 Infinity Auto-injector Chromatograph (G1329B) with a quaternary pump (G1311B/C), equipped with an Agilent 385 Evaporative Scattered Light Detector (Palo Alto, CA, USA). The chromatographic separation of the different reaction products obtained by enzymatic ethanolysis was carried out with a silica normal phase column (250 mm x 4.6 mm i.d., 5 μm) maintained at 30 °C [59] using a ternary gradient as follows: 0–2 min, 99.5% A and 0.5% B; at t = 6.5 min, 70% A and 30% B; at t = 11 min, 63% A, 27% B and 10% C; at t = 18 min, 99.5% A and 0.5% B; and at t = 20 min, and 99.5% A and 0.5% B. Eluent A consisted of 2,2,4- trimethylpentane, eluent B consisted of methyl tert-butyl ether, and eluent C consisted of 2-propanol.

The optimal signal and resolution of the ELSD detector were achieved with the following conditions: evaporator and nebulizer temperature of 30 °C, and evaporator gas (N_2_) at 1.6 SLM.

To identify different lipids represented in the sample such as TAG, diacylglycerols (DAG), monoacylglycerols (MAG), and FAEE, standards were individually injected and compared with standards already analyzed in previous studies.

### 3.8. Fractionation of Fatty Acid Ethyl Esters by Open-Column Chromatography

First, the filler material (6 g of silica) was added to the burette, as it has a glass frit in the lower part so that it remain fixed, adding the necessary hexane to dilute the silica to the burette. Then, more hexane was added to prevent the formation of bubbles in the column, and the sample was loaded into the column (0.3 g of the FAEE obtained from the scale-up of the enzymatic ethanolysis). To start separating the sample components in fractions (F), different mixtures of hexane and ethyl acetate were added to the column: 10 mL of hexane (F1); 10 mL of hexane–ethyl acetate (99:1) (F2); 10 mL of hexane–ethyl acetate (98:2) (F3); 15 mL of hexane–ethyl acetate (95:5) (F4); 5 mL of hexane–ethyl acetate (95:5) (F5); 5 mL of hexane–ethyl acetate (90:10) (F6) and another 5 mL of hexane–ethyl acetate (90:10) (F7). Experiments were made in duplicates. Fractions were kept refrigerated for GC-MS analysis.

### 3.9. Purification of an Enriched Dha Fraction by Open-Column Chromatography

The burette was prepared as in the previous section (see Section 3.8). The column was conditioned, and the sample was loaded (0.3 g of FAEE). Solvent mixtures to extract the first three fractions (P1, P2 and P3) were the same as in the previous protocol (see Section 3.8). Moreover, different mixtures were added: 10 mL of hexane–ethyl acetate (95:5) (P4); 5 mL of hexane–ethyl acetate (92:8) (P5); 5 mL of hexane–ethyl acetate (92:8) (P6); 5 mL of hexane–ethyl acetate (90:10) (P7); and 5 mL of hexane–ethyl acetate (90:10) (P8). Experiments were made in duplicate. Fractions were kept refrigerated for GC-MS analysis.

### 3.10. Analysis of Fatty Acid Composition by GC-MS

The fatty acid profile of the different fractions obtained by open-column chromatography was analyzed by GC-MS. Fractions were derivatized as reported in Section 3.5, and FAME were analyzed using GC-MS according to the described previously method. In this case, 200 µL of each fraction was collected on 400 µL of hexane for GC-MS analysis.

## 4. Conclusions

In conclusion, it should be noted that the use of alternative techniques of extraction from microalgal biomass, such as pressurized liquids, with subsequent enzymatic ethanolysis of the produced *Schizochytrium* oil, enables a sustainable and environmentally friendly procedure to effectively generate FAEE of DHA. Ethyl esters separated by column chromatography were fractionated and two objectives were achieved: On the one hand, an enriched fraction of DHA was obtained with complete recovery. On the other hand, it was possible to produce a fraction with high purity and the recovery of DHA, 93.2% and 86%, respectively, which may be used as a food supplement and to develop nutraceuticals and active pharmaceutical intermediates of marine origin in an integrated process.

## Figures and Tables

**Table 1:** Fatty acid profiles of different *Schizochytrium* sp. oils (commercial, extracted from biomass by PLE with different solvents and extracted from biomass using the Soxhlet method) analyzed by GC-MS. Microalgal oil 1 referred to *Schizochytrium* oil extracted with PLE using hexane. Microalgal oil 2 referred to *Schizochytrium* oil extracted with PLE using hexane–ethanol (1:1). Data were calculated as a percentage of fatty acid composition related to total ± standard deviation (SD).

	Commercial Oil	Microalgal Oil 1	Microalgal Oil 2	Soxhlet Extraction
14:0	-	10.41 ± 0.19	9.96 ± 0.45	12.72 ± 0.18
16:0	20.67 ± 0.04	20.30 ± 3.89	19.34 ± 0.57	23.02 ± 0.07
18:0	0.99 ± 0.02	1.15 ± 0.55	0.53 ± 0.09	0.41 ± 0.05
18:1 n-9	0.33 ± 0.09	0.80 ± 0.05	0.63 ± 0.03	0.64 ± 0.05
18:2 n-6	0.41 ± 0.03	0.27 ± 0.02	0.20 ± 0.07	0.14 ± 0.01
20:4	0.46 ± 0.08	0.81 ± 0.06	0.77 ± 0.04	0.83 ± 0.25
20:5 n-3 (EPA)	0.38 ± 0.02	0.98 ± 0.08	0.75 ± 0.04	0.83 ± 0.01
22:5 (DPA n-6)	15.42 ± 0.12	14.61 ± 0.58	15.49 ± 0.09	14.12 ± 0.30
22:5 (DPA n-3)	0.49± 0.04	0.36 ± 0.06	0.45 ± 0.03	0.33 ± 0.05
22:6 n-3 (DHA)	60.85 ± 0.32	49.25 ± 4.72	51.15 ± 0.72	45.58 ± 0.34

**Figure A1:**
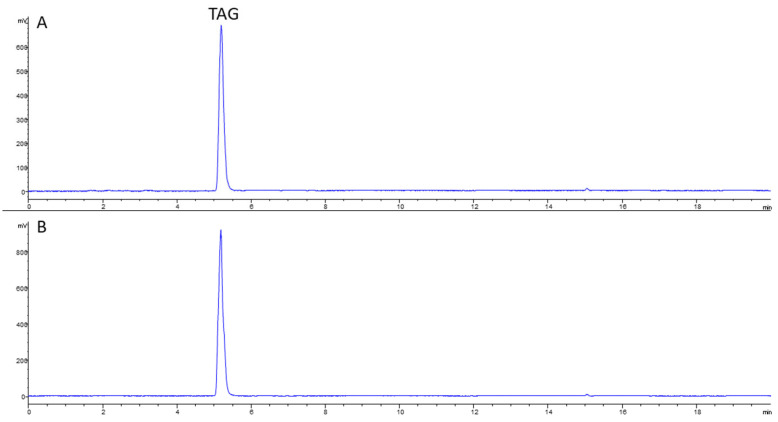
HPLC-ELSD analysis. Chromatograms correspond to the following: (**A**) microalgal oil 2; (**B**) commercial oil.

## Data Availability

The original data presented in the study are included in the article; further inquiries can be directed to the corresponding author.

## Appendix A

**Table A1:** Results obtained with the experimental design of PLE extraction. ^1^ 0 = 100% hexane; 50 = 50% hexane: 50% ethanol; 100 = 100% ethanol.

Temperature (°C)	Time (min)	Solvent ^1^	Extraction Yield (%)
80	10	0	21.85 ± 0.06
100	5	0	22.38 ± 0.34
100	15	0	22.62 ± 1.12
120	10	0	22.86 ± 0.45
80	5	50	26.11 ± 0.71
100	10	50	26.08 ± 0.13
100	10	50	25.87 ± 1.01
100	10	50	25.97 ± 1.23
80	15	50	25.71 ± 0.51
120	5	50	28.66 ± 0.78
120	15	50	29.06 ± 0.12
80	10	100	17.13 ± 1.17
100	5	100	21.41 ± 0.75
100	15	100	22.03 ± 1.85
120	10	100	24.81 ± 0.35

## Appendix C

To obtain the FAMEs in a basic medium, ISO TC34/SC 5 was followed:

− First, 25 mg of oil was mixed with 200 µL of hexane.
− Next, 50 µL of 2N KOH in methanol (prepared daily) was added.
− The mixture was shaken for 1 min in a vortex (Velp Scientifica ZX3).
− The mixture is rested for 5 min before the reaction proceeds.
− Then, 125 mg of sodium hydrogen sulfate monohydrate (NaHSO_4_ H_2_O) was added to stop the reaction and vortexed.
− Finally, the mixture was centrifuged for 5 min at 50 rpm (Hettich zentrifugen Mikro 120): Supernatant = FAMEs

For further analysis, 100 µL of FAMEs was collected from 400 µL of hexane.
